# From restrictive to more liberal: variations in moratlity among patients in opioid maintenance treament over a 12-year period

**DOI:** 10.1186/s12913-019-4382-9

**Published:** 2019-08-07

**Authors:** Anne Bukten, Marianne Riksheim Stavseth, Thomas Clasuen

**Affiliations:** Norwegian Centre for Addiction Research, University of Oslo, Kirkveien 166, N-0407 Oslo, Norway

**Keywords:** Overdose, Mortality, Opioid, Maintenance, Programme characteristics, Evaluation

## Abstract

**Background:**

As the effect of opioid maintenance treatment (OMT) on overdose mortality varies both between and within countries, treatment programs need to be evaluated in different treatment settings and over time within settings. We evaluated variations in mortality in a national programme: from the initial rollout as restrictive and low-capacity to its gradual change into more liberal and higher-volume.

**Methods:**

A 12-year prospective longitudinal cohort study including all persons (*n* = 6871) applying for and entering OMT in Norway (1997–2009). We followed all patients until 2009 or until death. We used crude mortality rates (CMR) to calculate overdose and all-cause mortality among patients in OMT before, during and after treatment, during a 12-year time-period. We also calculated variations in overdose and all-cause mortality over the course of treatment and after treatment termination. We fitted proportional hazards models with covariates to the data.

**Results:**

OMT significantly reduces risk of mortality compared to being outside of treatment. The reduction in overdose death was most substantial during the initial phase of the Norwegian OMT-programme, still; we consistently find that overdose deaths were more than halved in all calendar-periods throughout observation. We did not find an elevated risk of overdose death in the first weeks of treatment, nor in the first weeks after treatment cessation.

**Conclusion:**

In Norway, OMT reduces overall mortality. Reduction in mortality is likely dependent of both treatment delivery and characteristics of the at-risk population.

## Background

Although useful in clinical care, opioids are among the most harmful drugs in the world [1]. Untreated, opioid dependence is characterized as a chronic relapsing disorder associated with a number of negative outcomes, such as increased risk of infections (e.g. HIV or hepatitis) [1, 2], high rates of psychiatric and somatic comorbidities [3] and involvement in criminal activities [4, 5]. People with opioid dependence are nearly 15 times more likely to die than their peers [6], and drug overdose is the most common cause of death [1, 6]. The fact that deaths among people with opioid use disorders often occur at a young age [7], results in several decades of lost life per individual who succumb from an overdose.

Opioid use disorder is a complex health condition that often requires long-term treatment and care, and according to the World Health Organization, opioid agonist maintenance treatment often in terms of methadone and buprenorphine formulations, combined with psychosocial assistance, is the treatment of choice [8]. Following opioid withdrawal, naltrexone can help prevent relapse for patients who are motivated to abstain from opioid use [8, 9].

Today, opioid maintenance treatment (OMT) is a well-established treatment approach for opioid dependence and has proven effects on a number of outcomes including both social variables such as criminality [10] and health related variables such as morbidity [3]. Not least does several studies and meta-analysis agree that overdose mortality is reduced during OMT [11–15].

However, the risk of death for individuals with opioid use dependence seems to vary over the course of opioid maintenance treatment. Some studies have seen an increased risk in overdose death during the induction of treatment [14, 16–19], indicating that patients are especially vulnerable during periods of treatment transition [18]. In contrast to this, others have observed very low rates of mortality during the first 2 weeks of OMT [11, 20]. Differences in observations may be explained by differences in treatment approaches or characteristics of the at risk population, although this has not been sufficiently explored [21].

Globally, OMT is offered in a range of different ways [22]. Some programmes primarily provide medications, but limited additional psychosocial support [23] whereas other programmes invest large resources into psychosocial rehabilitation, including housing, social security benefits and treatment of comorbid disorders [24, 25]. Other variations pertain to prescribing doctors based either in primary care or in specialist centres [25–27], and at risk populations may vary according to mode of administration of opioids [25].

Furthermore, national OMT programs are varying across time regarding to which regulations and guidelines that at any given time defines treatment delivery [28]. The Norwegian treatment programme began as a restrictive programme with high thresholds for entry and low capacity [25]. When introduced in the late 90s, the OMT-programme were characterized by strict criteria for admission, based on severity and duration of opioid disorder, experience with abstinence oriented treatment, and age [25]. As the program became nationally widespread, there were gradual changes in how the original restrictive guidelines were practiced, and it gradually developed into a more liberal program with lower thresholds and expanded capacity to include more patients. The development of the programme performance in terms of capacity and priorities developed with time as the programme evolved. Norway thus provides an ideal setting for exploring the relationship between developments in OMT regulations and practices upon patient outcomes such as mortality.

As the effects of OMT is likely to be dependent both on the specifics of the program structure, the program quality as well as on the characteristics of the at-risk population [13, 21, 28], treatment programs need to be evaluated both over time and in different contexts, in order to settle how OMT is to be offered in the most appropriate manner and to improve our understanding of outcomes. On this background, we investigate mortality in a national cohort of patients in OMT over a 12-year period during which the Norwegian program went through different phases running from high threshold and low capacity to gradual liberalization and additional expansion. The aims of this study were to:

1. Describe patterns of treatment engagement and treatment retention during the 12-year observation period.
2. Investigate overdose and all-cause mortality among patients in OMT before, during and after treatment, in four different calendar time-periods (1997–1999, 2000–2002, 2003–2005 and 2006–2009)
3. Analyse variations in overdose and all-cause mortality over the course of treatment and after treatment termination (1–2 weeks, 2–4 weeks, 2–6 months and six months and after).

## Methods

### Study design

The study design is a prospective cohort study based on data from all patients included in OMT in Norway (*n* = 6871) in the period 01.01.1997–31.12.2009 linked to data from the Norwegian Cause of death registry in the same period. All data were linked through unique personal identification numbers (PINs).

### Setting

Compared to other countries, the Norwegian OMT program was introduced relatively late. Following several pilot-projects in the early 1990s, OMT was implemented as a national program in 1998 [24, 25]. At the time, treatment of substance use disorder with the use of medications was by many viewed with strong scepticism, as there was a strong belief in medication-free treatment. Consequently, the Norwegian criteria for admission to treatment was initially relatively strict: patients had to be at least 25 years old, diagnosed with opioid dependence and to have had undergone extensive abstinence oriented treatment [24]. As such, OMT was seen as a last resort type of treatment at the time, not the first choice of treatment as is the current practice.

In Norway, all inhabitants have equal access to health care and the OMT-programme is integrated into the general health and social security system. Patients apply for treatment via their General Practitioner or a social service centre. The programme can be described as universal as the same national guidelines apply for all centres [25].

The Norwegian OMT programme is based on WHO guidelines [8] and aims to be psychosocially oriented focusing on providing treatment for opioid dependence and social and health problems [22, 24]. Medications are to be used in order to support improved psychosocial functioning, like improved mental health and reduced substance use and criminal activity [25]. Treatment is offered long-term and often life-long. OMT is initiated in specialized treatment services in the secondary care level. At the specialized treatment services providing OMT, staff are specifically trained and experienced in the induction phase, related to the gradual introduction of medications and the adoption to ongoing prescription. As well as concurrent non-prescribed drug use. Treatment is generally started on the basis of a comprehensive action plan that includes gradual increasing agonist dosing, monitoring of survival determinants and measures in relation to the use of other drugs and social and health-related difficulties. Daily observed intake of medications is the rule during the induction of medications, and the psychosocial rehabilitation effort initially emphasise stable housing and access to social security benefits in addition do drug treatment counselling. Patients receiving treatment are to be offered social assistance in their local community [24].

### Data sources

#### The Norwegian opioid maintenance registry

The national OMT research registry was established based on patient records collected from each OMT centre in Norway. The OMT-registry includes both persons who applied for OMT, but never started, and all patents who entered OMT, at least once, in Norway in the period 1997–2009. More recent data than from 2009 were not available for this analysis. For research purposes, each centre provided lists of all patients including personal identification numbers (PINs). The register has overall high quality. Inconsistencies in the dataset (i.e. treatment stop before treatment start) were manually checked up against patients’ journals and corrected in the files.

#### The Norwegian cause of death registry

The Norwegian Cause of Death Registry includes complete death certificates reported by medical doctors after examination of the deceased. Death certificates are collected by the Norwegian Institute of Public Health, and include multiple ICD-10 causes of death [29] as well as information about both the underlying and immediate causes of death [30]. The coverage and the completeness of the Norwegian Death Registry is regarded as high as it comprises all residents and includes medical information on more than 98% of all deaths [30]. For cases of death with unknown cause or unexpected deaths, forensic autopsy including toxicology is the rule. Hence, reported data on overdose deaths are in most cases based on toxicological verification.

### Measures

The OMT registry (*n* = 7843) included some individuals (*n* = 972, of which 721 men) who were only registered with an application date, and had no date for treatment entry. Together with the remaining sample (*n* = 6871) who had at least one treatment episode, this group contributed to person-time in the pre-treatment period. The application-only group (*n* = 972) and the group who entered (n = 6871) were similar with respect to age at application for treatment (mean age 36 for both groups). The application-only group included some more men (*n* = 721, 74%) compared to the group who entered OMT (*n* = 4811, 70%).

Within the follow-up period, persons in the cohort often cycled in and out of treatment. Altogether 1650 individuals were registered with two or more treatment episodes, and contributed with time in the post-treatment period.

We defined following time at risk periods: *Pre-treatment*: the period between treatment application and death, between application and treatment start or between application and the end of observation (censored at 12 months). *In-treatment:* the period between treatment start and death, from treatment start to treatment stop or from treatment start and the end of observation. As the same patient may contribute to multiple observation periods and all treatment-periods are included in the analysis. *Post-treatment:* the period between treatment stop and death, from treatment stop to another treatment start, or from treatment stop to the end of the observation period. The same patient can contribute to several periods between treatment episodes, and all between-periods are included in the analysis.

Using ICD-10 codes we categorized causes of death into five mutually exclusive categories: ‘Overdose deaths’ (F11- F16, F19, F55, X40- X44, X60-X64, X85, Y10-Y14) (similar to EMCDDA’s “drug induced death indicator definition”), ‘accidents’ (V01-V99, W00-W19, W20-W99, X31, X00-X09, X58-X59), ‘suicide’ (X65- X84, Y87.0) and ‘cardiovascular disease/cancer’ (C00-C97, I00-I99, G45, G46). All other deaths were categorized as ‘other deaths’.

### Ethics

The study was approved by the Regional Committees for Medical and Health Research Ethics, Region South-East Norway, ref. no 2015/1257. The study did not require informed consent. Data linkage was performed by the Norwegian Institute of Public Health who prepared the files for analysis.

### Statistical analysis

Crude mortality rates (CMRs) and 95% confidence intervals were calculated as number of deaths per 1000 person years (PY) [31]. In order to compare crude mortality rates, rate ratios and 95% confidence intervals [32] were examined, with calculation of the ratio between two rates.

Potential factors associated with death during treatment and post-treatment were examined using Cox regression models. The coefficients were interpreted in terms of incidence hazard ratios (HR) with 95% confidence intervals. We ran univariate models for all covariates and one multivariate model.

## Results

A total of 6871 individuals were involved in 9038 treatment episodes during the 12-year study period. The number of annual treatment initiations increased progressively, from 1999 and onwards (Table 1). In the initial years, women comprised about 40% of all patients, but in later years, the proportion of women stabilized around 30%. Age of first treatment episode was stable at 37–38 years throughout the observation period. The 12-months retention rate per year of new treatment initiations (OMT naïve persons), declined somewhat during observation, but was stable at about 80% in the latter half of the observation period (Table 1).

**Table 1:** Number of treatment initiations and one-year retention rate per calendar year (*n* = 6871)

Year	Treatment initiations (n)	Men (%)	Mean age at first treatment initiation	One-year retention per year per treatment initiation (OMT naïve) (%)
1997	23	14 (61)	38	23 (100)
1998	141	88 (62)	38	124 (88)
1999	565	379 (67)	38	479 (85)
2000	459	303 (66)	37	384 (84)
2001	590	415 (70)	37	489 (83)
2002	682	478 (70)	37	478 (70)
2003	666	465 (70)	37	532 (80)
2004	630	456 (72)	36	530 (84)
2005	703	492 (70)	37	600 (85)
2006	661	471 (71)	37	537 (81)
2007	603	427 (71)	37	482 (80)
2008	506	349 (69)	36	411 (81)
2009	634	468 (74)	37	NA

During pre-treatment, the all-cause CMR was 19.7/1000 PY. In this period, overdose deaths accounted for 76% of all deaths with accidents accounting for six and suicide for 3% (Table 2). During treatment, the all-cause CMR was to 13.0/1000PY. Overdoses accounted for 42% of all deaths, accidents and suicide accounted for 4% respectively and cancer/cardiovascular for 16% of all deaths. During treatment, all-cause mortality was reduced to two thirds of pre-treatment levels (Rate Ratio 0.66, 95% CI 0.52–0.83) and overdose death was reduced to about one third (Rate Ratio 0.36, 95% CI 0.03–3.90) respectively. After treatment, overdose mortality accounted for almost 70% of all deaths, and the all-cause CMR was 33.7/10000PY.

**Table 2:** All cause and cause-specific mortality rates, before, during and after treatment; crude mortality rates (CMR) per 1000 person-years and 95% confidence interval

	Pre-treatment (*n* = 7843)			In treatment (n = 6871)			After treatment (*n* = 1650)		
	*n*	PY	CMR (CI)	*n*	PY	CMR (CI)	*n*	PY	CMR (CI)
Overdose	68	4526	15.0 (11.5–18.6)	160	29,172	5.5 (4.6–6.3)	159	6819	23.3 (19.7–26.9)
Non-overdose	21	4526	4.6 (2.7–6.6)	218	29,172	7.5 (6.5–8.5)	71	6819	10.4 (8.0–12.8)
Accidents	5	4526	1.1 (0.1–2.1)	15	29,172	0.5 (0.3–0.8)	8	6819	1.2 (0.4–2.0)
Suicide	3	4526	0.7 (0.0–1.4)	15	29,172	0.5 (0.3–0.8)	7	6819	1.0 (0.3–1.8)
Cancer/cardiovascular	3	4526	0.7 (0.0–1.4)	60	29,172	2.1 (1.5–2.6)	16	6819	2.3 (1.2–3.5)
Other	10	4526	2.2 (0.8–3.6)	128	29,172	4.4 (3.6–5.1)	40	6819	5.9 (4.0–7.7)
All-causes	89	4526	19.7 (15.6–23.8)	378	29,172	13.0 (11.7–14.3)	230	6819	33.7 (29.4–38.1)

Table 3 illustrates CMRs during pre-treatment, in-treatment and after treatment for four different calendar-periods (1997–1999, 2000–2002, 2003–2005 and 2006–2009). In the pre-treatment period, the all-cause mortality rate was particularly high during the first calendar-period (CMR 85.5/1000 PY), then reduced to less than one third during the second period (CMR 23.4/1000 PY) and gradually declined throughout calendar time. In pre-treatment, the majority of deaths in all calendar-periods were related to overdoses.

**Table 3:** All cause and overdose death before, during and after treatment during different calendar-periods (1997–1999, 2000–2002, 2003–2005 and 2006–2009); crude mortality rates (CMR) per 1000 person-years and 95% confidence interval

	Pre treatment			In treatment			After treatment		
	*n*	PY	CMR (CI)	*n*	PY	CMR (CI)	*n*	PY	CMR (CI)
1997–1999									
Overdose	21	316	66.5 (38.1–94.9)	25	4566	5.5 (3.3–7.6)	24	553	43.4 (26.1–60.8)
Non overdose	6	316	19.0 (3.8–34.2)	62	4566	13.6 (10.2–17.0)	21	553	38.0 (21.7–54.3)
All causes	27	316	85.5 (53.2–117.7)	87	4566	19.1 (15.1–23.1)	45	553	81.4 (57.6–105.2)
2000–20,002									
Overdose	23	1025	22.4 (13.3–31.6)	58	9197	6.3 (4.7–7.9)	66	1729	38.2 (29.0–47.4)
Non overdose	1	1025	1.0 (0–2.9)	78	9197	8.5 (6.6–10.4)	26	1729	15.0 (9.3–20.8)
All causes	24	1025	23.4 (14.0–32.8)	136	9197	14.8 (12.3–17.3)	92	1729	53.2 (42.3–64.1)
2003–2005									
Overdose	13	1061	12.3 (5.6–18.9)	53	9796	5.4 (4.0–6.9)	47	1919	24.5 (17.5–31.5)
Non overdose	7	1061	6.6 (1.7–11.5)	53	9796	5.4 (4.0–6.9)	17	1919	8.9 (4.6–13.1)
All causes	20	1061	18.9 (10.6–27.1)	106	9796	10.8 (8.8–12.9)	64	1919	33.4 (25.2–41.5)
2006–2009									
Overdose	11	1185	9.3 (3.8–14.8)	24	5613	4.3 (2.6–6.0)	22	2618	8.4 (4.9–11.9)
Non overdose	7	1185	5.9 (1.4–10.3)	25	5614	4.5 (2.7–6.2)	7	2618	2.7 (0.7–4.7)
All causes	18	1185	15.2 (8.2–22.2)	49	5614	8.7(6.3–11.2)	29	2618	11.1 (7.0–15.1)

In treatment, the all-cause mortality rates were declining from the first calendar-period and onwards (Table 3). The overdose mortality rates were stable in treatment (range CMR 5.5–4.3/1000 PY) across all periods. After treatment, mortality rates were high during the first calendar-period (CMR 81.4/1000 PY) and declined throughout calendar time. As in pre-treatment, the majority of deaths after treatment were related to overdoses (Table 3).

The Hazard rate for all-cause mortality both in-treatment and after treatment was somewhat higher in the first calendar-period compared to the later periods, but not a statistically significant difference (Table 4). Older age at treatment start was associated with higher risk for mortality during treatment (aHR 1.07, 95% CI 1.06–1.09). Women had reduced risk for mortality following treatment, compared to men (aHR 0.73, 95% CI 0.54–1.00).

**Table 4:** All-cause mortality by treatment period, by gender and age. Cox regression and 95% confidence intervals

	In-treatment (n = 6871)		After treatment (n = 1650)	
	HR (95% CI)	*P*-value	HR (95% CI)	*P*-value
Treatment period				
1997–1999 (reference)				
2000–2002	0.88 (0.67–1.18)	0.398	0.93 (0.34–2.57)	0.889
2003–2005	0.71 (0.52–0.97)	0.33	0,56 (0.20–1.56)	0.266
2006–2009	0.59 (0.39–0.98)	0.11	0.43 (0.51–1.20)	0.106
Gender				
Men (reference)				
Female	0.95 (0.76–2.20)	0.68	0.73 (0.54–1.00)	0.046
Age Treatment start	1.07 (1.06–1.09)	< 0,001	0.99 (0.97–1.01)	0.577

*HR* Hazard Ratio (estimated Hazard ratio for a one unit increase of change in the explanatory variable given the other variables held constant in the model)

During the entire 12-year observation period, we only observed two overdose deaths in the four weeks period following treatment induction and treatment termination respectively. In all phases following treatment, non-overdoses accounted for the majority of all deaths (Table 5). In the period following treatment, there was an increase in overdose-deaths after 2–6 months (CMR/1000PY 27.8, CI 17.5–38.1).

**Table 5:** Non-overdose and overdose death in different time-intervals following treatment start (n = 6871) and treatment cessation (n = 1650): crude mortality rates (CMR) per 1000 person-years and 95% confidence interval

	1–2 weeks			2–4 weeks			2–6 months			6 months and after		
	*n*	PY	CMR (CI)	*n*	PY	CMR (CI)	*n*	PY	CMR (CI)	*n*	PY	CMR (CI)
After treatment start												
Non-overdose	7	340	20.6 (5.3–35.8)	2	335	6.0 (0.0–14.2)	21	3124	6.7 (3.8–9.6)	348	28,894	12.0 (10.8–13.3)
Overdose	2	340	5.9 (0.0–14.0)	0	335	0	11	3124	3.5 (1.4–5.6)	147	28,894	5.1 (4.3–5.9)
After treatment stop												
Non-overdose	4	127	31.4 (0.6–62.2)	3	112	26.7 (0.0–56.9)	39	1008	38.7 (26.5–50.8)	184	6700	27.5 (23.5–31.4)
Overdose	0	127	0	2	112	17.8 (0.0–42.5)	28	1008	27.8 (17.5–38.1)	129	6700	19.3 (15.9–22.6)

## Discussion

The Norwegian OMT-programme consistently and significantly reduces risk of mortality compared to being outside of treatment among persons with opioid dependence. The reduction in overdose death was most substantial during the initial national roll-out of the Norwegian OMT-programme, in which the program was restrictive and had low capacity (1997–1999). Still, we consistently find that overdose deaths were more than halved throughout observation. We did not find an elevated risk of overdose death in the first weeks of treatment, nor in the first weeks after treatment cessation.

The variation in risk of mortality over calendar-time emphasize that one should take into consideration both changes in regulations of the programme, as well as differences in programme delivery across countries when monitoring the effect of OMT. Although systematic reviews of cohort studies can provide valuable evidence onto the mortality of opioid dependent people at different treatment periods of OMT [14], these studies should increasingly characterise programmes according to regulations and practices from which data were collected, in order to capture and categorize the programme performance better. In a recent meta-analysis, Sordo and colleagues [14] synthesised evidence from cohort studies on risk of mortality during and after opioid substitution treatment. The included studies were conducted in high-income countries, with the observation periods distributed across many years (1965–2010), and treatment provision likely varied across programs.

Similar to other countries like Sweden [28], the Norwegian OMT programme has evolved from high-threshold and low capacity to become more harm-reduction oriented high-volume programme [25]. The first period represents a situation in Norway when OMT had not been available and an escalating overdose crisis had emerged. Treatment priority was given to the most severely dependent and often critically ill persons. Given the strict guidelines at the time, all patients had more than 10 years of opioid addiction and multiple “failed” treatment episodes (non-medical) behind them [25]. In this initial phase, patients could also be excluded from treatment if they had a substantial misuse of other drugs, for example cannabis, during treatment. In our data, this period is reflected by high levels of pre-treatment overdoses, followed by a markedly reduction in overdoses within treatment, and then very high levels of overdose mortality among patients who terminated treatment. That mortality was most substantially reduced in this early period is therefore logical, as OMT was serving patients who had very high overdose risk and often many years of untreated illness behind them.

Gradual declines in pre-treatment overdose mortality is seen with more recent calendar-periods, which reflects a steady increase in treatment capacity and more people also with less critical illness were included (gradual lowering of threshold to treatment). The in-treatment overdose mortality remained stable and low across all periods, indicating the relatively stable protective effect that is achieved within the current setting within OMT also including “harm reduction” as a goal for OMT that represents the most recent phase.

Considering the fact that many patients are fluctuating in and out of treatment [10, 33], it is important that transition in treatment are taken into consideration when researchers are to investigate outcomes related to treatment. In contrast to earlier findings in other countries [14, 17], we did not observe an elevated risk of overdose death in the first weeks of treatment. This may be due to the way the treatment was provided [21]. In Norway OMT is initiated in specialized treatment centres at the secondary treatment level, and this treatment model seems to be efficient in delivering the treatment induction with high levels of safety; that is low risk of overdose, as opposed to what has been seen in other settings [17]. This indicates that OMT inductions preferably should be provided in settings where staff are used to and trained to deal with the complex task of OMT induction rather than in settings where induction of OMT may be a rare event to the clinician [21].

As opposed to other findings from other countries [17], our results did neither show high rates of mortality in the immediate period after cessation. This may be explained by a high tolerance to opioids developed as a patient in OMT, which gradually decreases over days and the first week following treatment cessation. Additionally data in the current dataset has detailed information on dates of medication termination and hence can define treatment termination specifically, maybe as opposed to other cohort studies.

During the entire observation period, we consistently observed higher mortality-rates for the group that terminated treatment compared to pre-treatment mortality levels. This may be explained at least in part by two factors; a selection of the group that terminates treatment being more severely affected by substance use and comorbidity compared to those who manage to remain in treatment, as well as ageing, which necessarily takes place for everyone with progression through the treatment system. In addition to this, it is likely that the situation associated to treatment interruption represent a period of crisis for the majority of patients discontinuing, making them all at particularly high risk of overdose in the weeks following treatment.

### Strengths and limitations

Our study includes a national cohort followed over a long period. As all Norwegian citizens are included in a universal health coverage, our cohort includes all patents, irrespective of their ability to pay for treatment. Few long-term studies evaluating OMT exist, and often only smaller samples and selected cohorts are included in the cohorts. As overdose deaths occur statistically infrequently, adequate population size and follow-up period is necessary to attain sufficient statistical strength. Moreover, we used nation-wide registers to ensure that no patients were lost to follow-up, making it practicable to address the possible effect of patients’ cycling in and out of treatment.

Some limitations must be taken into consideration when interpreting our results: no information on type of drugs used or prescribed medications were available, neither in the period before or during treatment. Nor whether termination of treatment was voluntary or not. However, gradually towards 2009 the practice of involuntary treatment termination ended. Unfortunately, the dataset included no information about individual type of maintenance medication or dosage that included both methadone and buprenorphine formulations. However, in 2009 mean dosing of methadone around 100 mg daily and 18 mg for buprenorphine. It may also be noted that the lack of overdoses observed in the first weeks after treatment start/treatment stop, may be due to relatively few observed deaths (in statistical terms) during these short time periods and thus lower statistical power.

Another limitation is that the study follow-up ended in 2009, as data was no longer available for the complete treatment population as included in these analyses. Despite being somewhat dated, the description of a national treatment program over a 12 year period, and especially in a time of change, is relevant to date. OMT is a type of treatment that must be continuously adapt to meet the needs of patients, adhere to politics and to general changes in how different substance use disorders are treated.

## Conclusion

Our results demonstrate that mortality in the at-risk population entering the Norwegian OMT changed with calendar-time, and was particularly high during the initial years when the OMT-programme was introduced and had limited capacity. The in-treatment overdose mortality was stable and low, and there was no peak in overdose mortality during the first weeks while induction to medications. It is likely that an integrated treatment approach, combining psychosocial support with pharmacological therapy as well as treatment delivery and initiation in specialized centres at the secondary care level is protective of overdose mortality risk during treatment induction.

For future research into OMT-cohorts, awareness of programme delivery and type when examining differences in outcomes is essential for understanding and improving treatment effectiveness. It is important to monitor outcomes according to the way treatment is offered over calendar-time, as treatment programmes may shift from high to more low-threshold, by way of both Sweden and Norway have experienced. It is also likely that the ratio of treatment benefit is associated to the situation and context when treatment is given; a low-threshold programme combined with less treatment demand and support will probably result in lower reductions of mortality compared with high-threshold programmes, where the most severely ill are given priority.

## Data Availability

The datasets used and/or analysed during the current study are available from the corresponding author on reasonable request.

## Abbreviations

CMR: Crude mortality rate
OMT: Opioid maintenance treatment
PIN: Personal identification number
PY: Person years
